# Urinary metabolomics data and cognitive performance in an adult population

**DOI:** 10.1007/s11011-026-01977-8

**Published:** 2026-09-12

**Authors:** Annika Kneipp, Inge Kirchberger, Dennis Freuer, Christine Meisinger, Jakob Linseisen

**Affiliations:** Institute of Epidemiology, Faculty of Medicine, University of Augsburg, Stenglinstraße 2, Augsburg, 86156 Germany

**Keywords:** Cognition, Executive function, Digit span, Verbal fluency, Urinary metabolites, Metabolomics

## Abstract

With an ageing population, the social and economic burden of cognitive decline is increasing. Urinary metabolomics may offer a promising approach for identifying metabolic markers for early cognitive impairment and gaining insights into underlying biological mechanisms. The aim of this study was to investigate the association between urinary metabolites and cognitive performance in a population-based sample. Cognitive performance was assessed in 560 (digit span) and 553 participants (semantic verbal fluency) of the *Metabolism*,* Nutrition and Immune System in Augsburg (MEIA)* study. Urinary concentrations of 51 metabolites were quantified via ¹H nuclear magnetic resonance and normalized to creatinine (Nightingale Health, Finland). Overall and sex-specific associations between metabolites and cognitive outcomes were examined using multivariable linear regression models adjusted for demographic, lifestyle, and health-related confounders. Five metabolites were significantly associated with digit span performance: 3-hydroxyhippurate, trans-aconitate, and uracil in the overall sample, and taurine and pyroglutamate in men only. Eight metabolites were associated with verbal fluency, six of which were observed in the full cohort, namely, acetate, citrate, hippurate, pyroglutamate, taurine, and xanthosine, while glutamine showed associations exclusively in men and propylene glycol exclusively in women. After adjustment for multiple testing, none of the associations remained significant. Urinary metabolites related to energy, microbial, amino acid, and nucleotide metabolism showed domain- and sex-specific associations with cognitive performance. These exploratory findings point towards a potential involvement of systemic metabolic processes related to mitochondrial energy production, oxidative stress, inflammation, and neurotransmission, during early cognitive changes in the general population.

**Supplementary Information:**

The online version contains supplementary material available at https://doi.org/10.1007/s11011-026-01977-8.

## Introduction

Cognitive function is a key determinant of individuals’ health, independence, and overall well-being. Impairments in cognitive performance are associated with increased mortality and represent a growing public health concern in ageing populations (Pavlik et al. 2003). With rising life expectancy worldwide, the prevalence of cognitive decline is expected to grow substantially, with current estimates indicating that the number of individuals affected by dementia will triple by 2050 (GBD 2019 Dementia Forecasting Collaborators 2022). This imposes a serious burden on individuals and society, while effective strategies for early detection and prevention remain limited (Fang et al. 2025).

The most common form of dementia is Alzheimer’s disease (AD), accounting for an estimated 70% of all cases (Alzheimer's Association 2024). In AD, cognitive decline typically develops gradually over many years, with subtle impairments already detectable in the preclinical stage, prior to onset of mild cognitive impairment (MCI) (Wilson et al. 2011). Executive functions, including working memory, cognitive flexibility, and inhibitory control are particularly sensitive to these early changes in cognitive health (Guarino et al. 2018).

Recent research suggests that cognitive aging and neurodegenerative processes are accompanied by substantial metabolic alterations, which can be captured using high-throughput metabolomics technologies (Panyard et al. 2022). By comprehensively characterizing small-molecule metabolites that reflect genetic, environmental, and lifestyle influences, metabolomics has become a key tool for biomarker discovery and pathway analysis (Johnson et al. 2016).

There are some studies investigating metabolomics in relation to cognitive performance in the general population, however, most of them have focused on blood samples or cerebrospinal fluid (CSF) (Ahmad 2024; Bressler et al. 2017; Shi et al. 2019), while urine remains a promising, yet comparatively underexplored biofluid (Seol et al. 2020). Unlike blood and CSF, urinary metabolites are less tightly regulated by homeostatic mechanisms and may therefore capture more subtle systemic metabolic changes (Wu and Gao 2015). To date, urinary metabolomics studies in the context of cognition have largely been limited to clinical populations, particularly individuals with dementia (Kurbatova et al. 2020; Yilmaz et al. 2020; Zhang et al. 2019), whereas potential early or subtle impairments in individuals from general populations remain insufficiently studied.

To the best of our knowledge, this is among the first studies to investigate the association between urinary metabolites and cognitive function in a general population sample, with a specific focus on executive function as an early marker of cognitive decline, assessed through digit span performance and semantic verbal fluency.

## Materials & methods

### Study sample

The present study uses data from the population-based *Metabolism*,* Nutrition and Immune System in Augsburg *(MEIA) study, which has been described in detail elsewhere (Kneipp et al. 2026).

A random sample of 594 adults aged 18–75 years was recruited and examined between April 2021 and July 2023. For the present analyses, 11 participants were excluded due to missing urinary metabolite measurements and an additional 23 participants due to missing data on both cognitive tests, resulting in a final sample of 560 participants for the digit span analysis. Further, seven participants did not complete the semantic verbal fluency task, yielding a sample of 553 for this outcome.

### Data collection

Data collection took place at the study centre of the Institute of Epidemiology, University Hospital Augsburg. Participants completed a standardized, computer-assisted questionnaire covering demographic information, lifestyle factors, and health conditions. Collected variables included sex, age, educational attainment, smoking, risky alcohol consumption, and physical activity. Self-reported health conditions encompassed psychiatric disorders, including depression, anxiety and psychosis, major cardiovascular events, such as myocardial infarction and stroke, and fatigue symptoms.

During study centre visits, participants also underwent a series of physical, cognitive, and anthropometric assessments (including body weight and height), and provided biospecimens including fasting venous blood, spot urine, and fecal samples. For the present study, metabolomic profiling was performed on spot urine samples.

### Cognitive assessment

Two key domains of cognitive performance, particularly executive function, were assessed in this study: Semantic verbal fluency, using a category fluency task, and working memory, using the digit span test.

#### Digit span

Digit span is a subtest of the Wechsler Adult Intelligence Scale-IV (WAIS-IV) and, as part of its Working Memory Index (WMI), evaluates working memory capacity, a core component of executive functioning (Egeland et al. 2026). The test comprises three conditions: Forward, backward, and sequencing digit span. In the forward condition, participants verbally repeat number sequences in the order presented, whilst in the backward condition, sequences must be recalled in reverse order. The sequencing condition requires the rearrangement of digits in ascending numerical order (Wechsler 2008).

A total digit span score was calculated as the sum of all three conditions and age-corrected scaled scores (range: 1–19) were used for the present analyses. These standardized scores are derived from raw performance scores and adjusted according to age-specific norms, with a mean of 10 and a standard deviation of 3, allowing for comparability across different age groups. For descriptive analyses, scaled scores were additionally categorized as “below average" (1-6), "average" (7-13), and “above average” (14-19) (Wechsler 2008).

#### Verbal fluency

Semantic verbal fluency was assessed using a category fluency task based on the Regensburg Verbal Fluency Test (Regensburger Wortflüssigkeitstest, RWT) (Aschenbrenner et al. 2000), a standardized German neuropsychological instrument equivalent to the English Controlled Oral Word Association Test (COWAT) (Ross et al. 2007). Participants were instructed to name as many animals as possible within two minutes and performance was quantified as age-adjusted percentile ranks. For descriptive analyses, percentile ranks were categorized as “below average” (1st-15th), “average” (16th-84th), and “above average” (85th-99th). This task reflects language abilities as well as executive functions, particularly lexical retrieval processes and semantic memory (Baldo et al. 2006; Shao et al. 2014).

### Metabolomics analysis in urine samples

Spot urine samples (20 mL) were obtained from participants in the morning hours during their study centre visit, following an overnight fast of at least 12 h. Samples were centrifuged, aliquoted, and stored at − 80 °C until metabolomic analysis. Profiling of 50 urinary metabolites plus creatinine was conducted by Nightingale Health Ltd. using a high-throughput ¹H nuclear magnetic resonance (¹H NMR) spectroscopy platform with automated total-line-shape fitting (Bizzarri 2023; Mutter et al. 2022).

To account for dilution effects, metabolite concentrations were normalized to urinary creatinine and subsequently scaled by a factor of 100 to improve interpretability (Li et al. 2022). Values below the limit of detection were imputed as half of the lowest observed concentration for the respective metabolite, corresponding to the analytical detection limit.

Detailed descriptions of sample processing, NMR analysis, metabolite quantification, and quality control procedures are provided elsewhere (Kneipp et al. 2026).

### Confounders

Confounders were identified based on existing literature and comprised demographic characteristics, lifestyle factors, and health conditions relevant to cognitive function. Demographic variables included age (years), sex, and body mass index (BMI, kg/m²), calculated from measured body weight and height. Age was included as a continuous variable in all regression analyses, while participants were additionally grouped into the age categories “young” (18–39 years), “middle-aged” (40–60 years), and “older adults” (61–75 years) for descriptive purposes. Educational attainment was categorized according to the International Standard Classification of Education (ISCED; levels 1–3 and 4–6) (OECD 1999).

Lifestyle factors encompassed physical activity, smoking status (“never,” “former,” or “current”), and alcohol consumption. Physical activity was evaluated using the European Health Interview Survey-Physical Activity Questionnaire (EHIS-PAQ), capturing frequency, duration, and intensity of activity, and categorized into four levels (“sedentary,” “low active,” “active,” and “very active”) (Finger et al. 2015; Gerrior et al. 2006). Risky alcohol intake was assessed using the Alcohol Use Disorders Identification Test (AUDIT) and categorized into four groups based on sex-specific cut-offs: low (men 0–3, women 0–2), moderate (men 4–5, women 3–5), high (both sexes 6–7), and severe (both sexes 8–12) (Saunders et al. 1993).

Health-related variables included conditions associated with cognitive impairment, namely psychiatric disorders (self-reported depression, anxiety, or psychosis) and major cardiovascular diseases (CVD; stroke or myocardial infarction). In addition, fatigue was considered as a potential confounder and assessed using the Fatigue Assessment Scale (FAS), applying a cut-off of 22 (FAS < 22: no fatigue; ≥ 22: fatigue) (Lynch et al. 2007).

### Statistical analysis

Categorical variables are described as absolute and relative frequencies, while continuous variables are summarized using medians and 25th and 75th percentiles. Group differences were evaluated using Chi-square tests for categorical variables and Kruskal-Wallis tests for continuous variables. Corresponding *p*-values < 0.05 were considered statistically significant.

Of the 51 quantified urinary metabolites, 46 were retained for the analyses. Creatinine was applied exclusively for normalization purposes and was therefore not treated as an independent variable and four metabolites (3-hydroxyisovalerate, ethanol, 2-furoylglycine, and glucose) were excluded due to highly skewed distributions, limited variability, or a high proportion of zero values.

Associations between urinary metabolites and cognitive performance were examined using multivariable linear regression models. The two primary cognitive outcomes, semantic verbal fluency and digit span, were analysed separately as continuous variables. Despite the exploratory nature of the study, false discovery rate (FDR) adjustment was applied to the *p*-values obtained from the metabolite-cognition association analyses to account for multiple testing. Based on prior literature, regression models were adjusted for age, sex, BMI, educational attainment, physical activity, smoking status, and alcohol consumption. In addition, relevant health conditions were included, namely psychiatric disorders (depression, anxiety, psychosis), CVD (stroke and myocardial infarction), and fatigue.

Model assumptions were evaluated and linearity between the continuous variables age, BMI and urinary metabolites and the cognitive outcomes was assessed using second-degree polynomial terms. Where deviations from linearity were observed, restricted cubic splines with three knots were applied.

Potential effect modification by age, sex, and BMI was assessed for all metabolites. In the presence of statistically significant interactions, stratified analyses were conducted.

Analyses were primarily conducted in SPSS (version 29.0.0). Spline regression analyses and figure preparation were performed in R (version 4.5.1).

## Results

### Descriptive data

Baseline characteristics of the study population are presented in Table 1 for the total sample (*n* = 553) and stratified by performance in the verbal fluency task. The sample comprised slightly more women (56.1%) than men (43.9%). The median age of the study population was 49.0 years (34.0; 60.0), with the majority of participants being middle-aged adults (41.4%), followed by young adults (34.4%) and older adults (24.2%). In total, 47.6% of the participants showed average performance in the verbal fluency test, whereas 10.3% performed below average and 42.1% above average. Sex distribution and smoking status differed significantly across verbal fluency performance groups (*p* < 0.001 and *p* = 0.004, respectively), with a higher proportion of females and never-smokers in the above-average group. Digit span performance also differed significantly across verbal fluency performance groups, with lower median scores in the below-average and higher scores in the above-average verbal fluency group (*p* < 0.001). In contrast, no significant differences were found with regard to cognition-related health conditions.

**Table 1:** Baseline characteristics of participants of the population-based MEIA study, stratified by verbal fluency (percentile rank, RWT)

	Total (*n* = 553)	Below Average (*n* = 57)	Average (*n* = 263)	Above Average (*n* = 233)	*p*-values	N*
Median (Q25, Q75)						
Age [years]	49.0 (34.0; 60.0)	47.0 (33.0; 54.0)	44.0 (32.0; 60.0)	54.0 (39.0; 61.0)	< 0.001	553
BMI [kg/m^2^]	25.7 (22.8; 29.0)	26.4 (24.2; 29.9)	25.8 (22.7; 29.3)	25.4 (22.5; 28.7)	0.586	552
Digit Span (WAIS-IV)	9 (8; 11)	8 (6; 10)	9 (7; 11)	10 (8; 12)	< 0.001	553
*n (%)*						
Sex					< 0.001	553
Female	310 (56.1%)	18 (31.6%)	143 (54.4%)	149 (63.9%)		
Male	243 (43.9%)	39 (68.4%)	120 (45.6%)	84 (36.1%)		
Age					0.001	553
18–39 years	190 (34.4%)	21 (36.8%)	108 (41.1%)	61 (26.2%)		
40–60 years	229 (41.4%)	29 (50.9%)	91 (34.6%)	109 (46.8%)		
61–75 years	134 (24.2%)	7 (12.3%)	64 (24.3%)	63 (27.0%)		
Education level					0.507	553
ISCED 1–3	110 (19.9%)	14 (24.6%)	54 (20.5%)	42 (18.0%)		
ISCED 4–6	443 (80.1%)	43 (75.4%)	209 (79.5%)	191 (82.0%)		
Physical activity					0.583	549
Sedentary	107 (19.5%)	7 (12.3%)	56 (21.5%)	44 (19.0%)		
Low active	166 (30.2%)	19 (33.3%)	76 (29.1%)	71 (30.7%)		
Active	157 (28.6%)	14 (24.6%)	75 (28.7%)	68 (29.4%)		
Very active	119 (21.7%)	17 (29.8%)	54 (20.7%)	48 (20.8%)		
Smoking status					0.004	553
Current	93 (16.8%)	13 (22.8%)	57 (21.7%)	23 (9.9%)		
Never	274 (49.5%)	28 (49.1%)	117 (44.5%)	129 (55.4%)		
Previous	186 (33.6%)	16 (28.1%)	89 (33.8%)	81 (34.8%)		
Risky alcohol consumption					0.119	550
Low	266 (48.4%)	28 (50.0%)	125 (47.7%)	113 (48.7%)		
Moderate	209 (38.0%)	14 (25.0%)	104 (39.7%)	91 (39.2%)		
High	54 (9.8%)	9 (16.1%)	23 (8.8%)	22 (9.5%)		
Severe	21 (3.8%)	5 (8.9%)	10 (3.8%)	6 (2.6%)		
Psychiatric disorder					0.498	551
Yes	55 (10.0%)	7 (12.3%)	22 (8.4%)	26 (11.2%)		
No	496 (90.0%)	50 (87.7%)	239 (91.6%)	207 (88.8%)		
CVD					0.264	552
Yes	19 (3.4%)	4 (7.0%)	7 (2.7%)	8 (3.4%)		
No	533 (96.6%)	53 (93.0%)	255 (97.3%)	225 (96.6%)		
Fatigue					0.342	548
Yes (FAS ≥ 22)	129 (23.5%)	12 (21.1%)	68 (26.4%)	49 (21.0%)		
No (FAS < 22)	419 (76.5%)	45 (78.9%)	190 (73.6%)	184 (79.0%)		
Digit Span (WAIS-IV)					< 0.001	553
Below average	69 (12.5%)	18 (31.6%)	39 (14.8%)	12 (5.2%)		
Average	451 (81.6%)	39 (68.4%)	214 (81.4%)	198 (85.0%)		
Above average	33 (6.0)	0 (0.0%)	10 (3.8%)	23 (9.9%)		

*Number of cases with valid information

*P*-values were derived from Kruskal-Wallis tests for continuous variables and Chi-Square tests for categorical variables

Psychiatric disorders include self-reported depression, anxiety, and psychosis

CVD include major cardiovascular diseases, such as stroke and myocardial infarction

Supplementary Table S1 summarizes baseline characteristics for participants with available data on digit span performance (*n* = 560), both overall and stratified by performance level. In this domain, 81.1% of participants showed average performance, 6.1% performed above average, and 12.8% below average. Baseline characteristics were largely comparable across performance groups, except for age, which was highest in the above-average group with a median of 57.0 years (48.8; 64.3).

Table 2 summarizes urinary metabolite concentrations (mmol/mmol creatinine × 100) for the overall sample and stratified by verbal fluency performance. Several metabolites exhibited significant differences across groups, including citrate, dimethylamine, hippurate, pyroglutamate, pseudouridine, quinic acid, xanthosine (all *p* < 0.001), glutamine (*p* = 0.034), 3-(3-hydroxyphenyl)-3-hydroxypropionic acid (*p* = 0.032), indoxyl sulfate (*p* = 0.029), mannitol (*p* = 0.049), taurine (*p* = 0.008) and trigonelline (*p* = 0.005).

**Table 2:** Creatinine-corrected urinary metabolite concentrations [mmol/mmol creatinine x 100] in the total study population and stratified by verbal fluency performance goups (RWT)

	Total (*n* = 553)	Below Average (*n* = 57)	Average (*n* = 263)	Above Average (*n* = 233)	*p*-values^a^
Acetate	0.466 (0.263; 0.794)	0.400 (0.236; 0.651)	0.438 (0.245; 0.779)	0.511 (0.295; 0.913)	0.076
Alanine	1.764 (1.334; 2.246)	1.542 (1.184; 2.090)	1.804 (1.353; 2.342)	1.747 (1.341; 2.136)	0.162
Allantoin	0.544 (0.273; 0.965)	0.504 (0.243; 0.872)	0.560 (0.286; 0.988)	0.544 (0.265; 0.971)	0.420
2-Hydroxyisobutyrate	0.495 (0.402; 0.592)	0.495 (0.375; 0.556)	0.477 (0.385; 0.583)	0.507 (0.417; 0.600)	0.092
Arabinose	0.443 (0.301; 0.645)	0.462 (0.288; 0.687)	0.427 (0.292; 0.628)	0.461 (0.318; 0.657)	0.258
3-Aminoisobutyrate	0.498 (0.159; 1.133)	0.393 (0.157; 0.860)	0.542 (0.158; 1.312)	0.480 (0.166; 1.026)	0.617
3-Hydroxyisobutyrate	0.683 (0.521; 0.874)	0.675 (0.470; 0.905)	0.681 (0.517; 0.878)	0.704 (0.530; 0.870)	0.718
Cis-Aconitate	1.717 (1.386; 2.216)	1.680 (1.274; 2.216)	1.713 (1.379; 2.279)	1.740 (1.401; 2.180)	0.818
Citrate	19.135 (11.846; 29.033)	12.234 (7.339; 21.847)	18.099 (11.200; 26.446)	22.410 (13.841; 30.979)	< 0.001*
Dimethylamine	2.964 (2.707; 3.305)	2.688 (2.510; 3.044)	2.951 (2.704; 3.338)	3.044 (2.787; 3.301)	< 0.001*
4-Deoxyerythronic acid	0.684 (0.528; 0.912)	0.631 (0.472; 0.913)	0.716 (0.539; 0.914)	0.674 (0.526; 0.907)	0.374
4-Deoxythreonate	2.095 (1.556; 2.720)	2.095 (1.606; 2.795)	2.030 (1.507; 2.742)	2.115 (1.596; 2.710)	0.705
Ethanolamine	3.992 (3.024; 5.064)	3.779 (3.008; 4.678)	3.992 (3.003; 4.965)	4.042 (3.062; 5.231)	0.452
Formate	1.516 (1.006; 2.003)	1.493 (0.928; 2.134)	1.486 (1.010; 1.989)	1.538 (0.999; 1.979)	0.999
Glutamine	2.022 (1.140; 3.159)	1.729 (0.858; 2.759)	2.202 (1.277; 3.397)	1.893 (1.061; 2.996)	0.034*
Glycine	8.059 (5.534; 11.485)	6.895 (5.187; 9.800)	8.018 (5.534; 11.984)	8.258 (5.686; 11.170)	0.193
Glycolic acid	3.887 (2.758; 5.243)	3.886 (2.783; 5.245)	4.055 (2.881; 5.443)	3.717 (2.693; 5.043)	0.109
Hippurate	23.687 (14.818; 39.009)	20.542 (14.818; 30.972)	20.879 (12.911; 35.618)	28.171 (17.312; 45.739)	< 0.001*
3-(3-Hydroxyphenyl)-3-hydroxypropionic acid	1.461 (0.483; 3.060)	1.130 (0.026; 2.601)	1.400 (0.483; 2.830)	1.657 (0.681; 3.467)	0.032*
Hypoxanthine	0.816 (0.591; 1.117)	0.746 (0.595; 1.029)	0.785 (0.544; 1.089)	0.881 (0.629; 1.138)	0.052
Isoleucine	0.086 (0.056; 0.139)	0.076 (0.053; 0.136)	0.090 (0.056; 0.141)	0.085 (0.056; 0.136)	0.657
Indoxyl Sulfate	2.528 (1.784; 3.476)	2.109 (1.572; 3.256)	2.392 (1.733; 3.407)	2.700 (1.977; 3.622)	0.029*
Lactate	0.898 (0.586; 1.524)	0.809 (0.580; 1.436)	0.904 (0.603; 1.622)	0.922 (0.578; 1.461)	0.371
Leucine	0.173 (0.134; 0.224)	0.181 (0.134; 0.229)	0.174 (0.137; 0.233)	0.170 (0.129; 0.209)	0.454
Mannitol	1.039 (0.418; 3.170)	0.918 (0.337; 2.691)	0.930 (0.382; 2.716)	1.297 (0.489; 4.119)	0.049*
3-Hydroxyhippurate	1.456 (0.733; 2.927)	1.160 (0.438; 2.346)	1.360 (0.770; 2.628)	1.645 (0.741; 3.174)	0.107
1-Methylnicotinamide	0.620 (0.460; 0.839)	0.665 (0.505; 0.839)	0.626 (0.428; 0.843)	0.614 (0.478; 0.822)	0.693
Pyroglutamate	2.245 (1.913; 2.650)	2.044 (1.707; 2.417)	2.223 (1.873; 2.568)	2.333 (1.995; 2.812)	< 0.001*
4-Hydroxyhippurate	1.056 (0.735; 1.556)	1.001 (0.673; 1.364)	1.086 (0.774; 1.640)	1.048 (0.715; 1.500)	0.137
Propylene glycol	0.366 (0.187; 0.613)	0.282 (0.156; 0.701)	0.369 (0.167; 0.603)	0.372 (0.204; 0.631)	0.551
Proline betaine	0.630 (0.305; 1.486)	0.610 (0.264; 1.650)	0.630 (0.310; 1.461)	0.659 (0.306; 1.491)	0.919
Pseudouridine	3.027 (2.740; 3.315)	2.752 (2.466; 2.970)	3.014 (2.725; 3.355)	3.077 (2.818; 3.321)	< 0.001*
Quinic acid	2.219 (1.065; 3.773)	1.476 (0.424; 2.831)	2.048 (1.051; 3.625)	2.631 (1.306; 4.072)	< 0.001*
Sucrose	0.051 (0.000; 0.227)	0.026 (0.000; 0.124)	0.058 (0.000; 0.225)	0.043 (0.000; 0.234)	0.285
Trans-Aconitate	0.433 (0.323; 0.545)	0.446 (0.325; 0.526)	0.425 (0.322; 0.562)	0.433 (0.324; 0.539)	0.993
Taurine	2.844 (0.573; 6.423)	3.647 (1.478; 7.722)	3.398 (0.590; 6.683)	2.157 (0.387; 5.323)	0.008*
Threonine	0.584 (0.389; 0.833)	0.581 (0.406; 0.769)	0.606 (0.401; 0.844)	0.560 (0.368; 0.829)	0.336
Trimethylamine-N-oxide	3.546 (2.504; 5.136)	3.220 (2.172; 4.768)	3.517 (2.319; 5.346)	3.611 (2.702; 5.082)	0.241
Trigonelline	2.900 (1.464; 4.658)	2.123 (0.964; 3.816)	2.625 (1.389; 4.252)	3.263 (1.786; 5.374)	0.005*
Tryptophan	0.560 (0.406; 0.739)	0.525 (0.404; 0.653)	0.580 (0.404; 0.747)	0.536 (0.411; 0.737)	0.406
Tyrosine	0.937 (0.653; 1.282)	0.927 (0.684; 1.216)	0.993 (0.685; 1.356)	0.916 (0.610; 1.199)	0.276
Uracil	0.518 (0.389; 0.679)	0.505 (0.373; 0.622)	0.515 (0.382; 0.669)	0.533 (0.423; 0.737)	0.180
Urea	3226.875 (2410.883; 4118.812)	3103.860 (2206.475; 3820.077)	3197.238 (2313.336; 4280.697)	3326.280 (2572.953; 4147.288)	0.202
Valine	0.217 (0.166; 0.276)	0.226 (0.161; 0.281)	0.226 (0.165; 0.297)	0.205 (0.168; 0.263)	0.128
Xanthosine	0.886 (0.783; 1.015)	0.806 (0.701; 0.932)	0.876 (0.778; 1.024)	0.910 (0.808; 1.020)	< 0.001*
Xylose	0.602 (0.353; 0.802)	0.558 (0.355; 0.689)	0.580 (0.344; 0.816)	0.644 (0.372; 0.858)	0.056

* *p* < 0.05

^a^ Kruskal-Wallis Test

Differences across digit span performance groups were found only for leucine (*p* = 0.038; Supplementary Table S2).

### Associations between urinary metabolites and cognition

#### Digit span

**Fig. 1:**
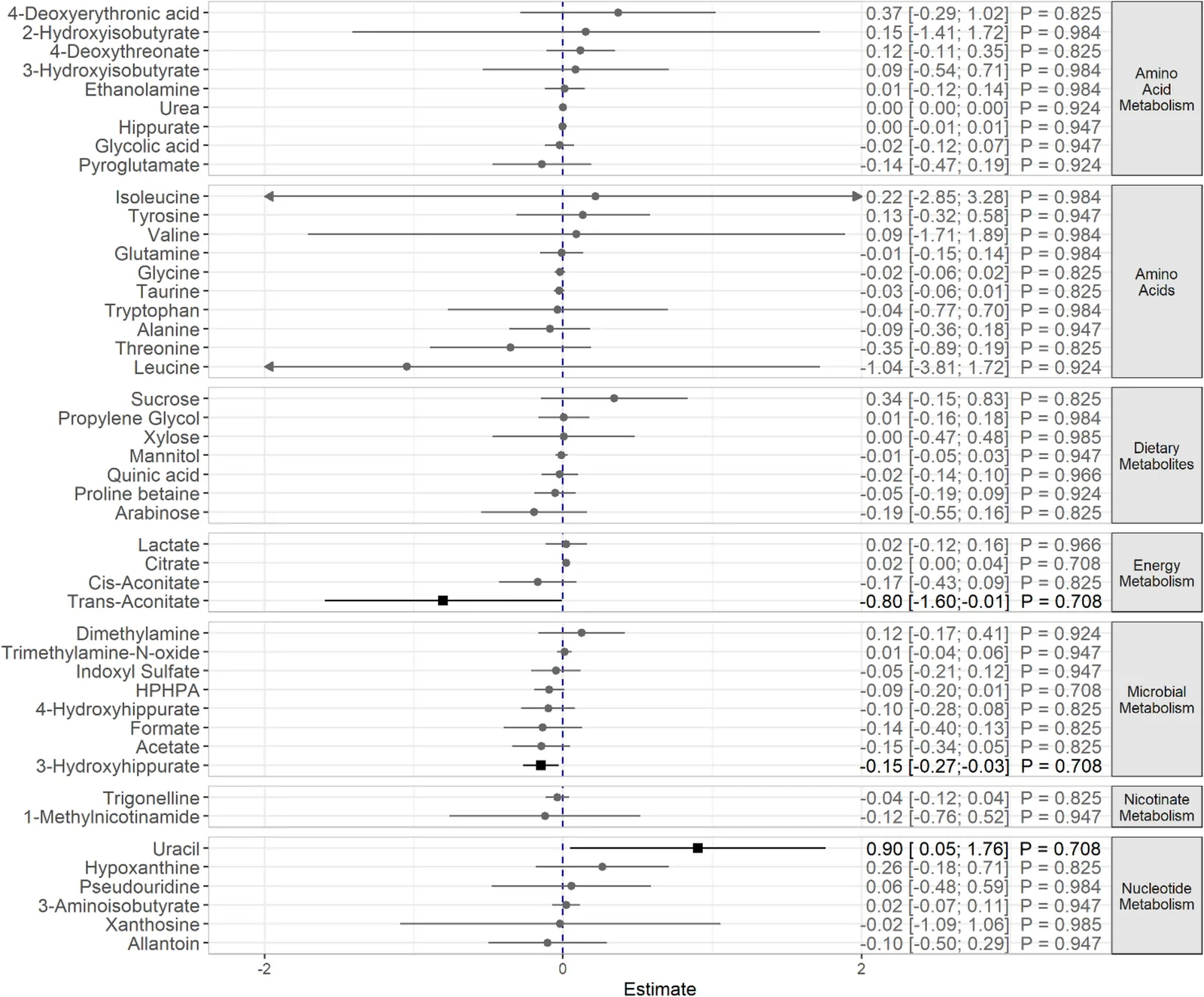
Associations between urinary metabolites and digit span adjusted for age, sex, BMI, education level, physical activity level, smoking status, alcohol consumption, and cognition-related health conditions (psychiatric diseases, CVD and fatigue). Forest plots display regression coefficients and 95% confidence intervals. Corresponding *p*-values after FDR correction are presented on the right. Squares indicate associations that were statistically significant before FDR correction. Metabolites were grouped according to metabolic pathways. HPHPA: 3-(3-Hydroxyphenyl)-3-hydroxypropionic acid

As depicted in Fig. 1, significant associations with the digit span score were observed for 3-hydroxyhippurate (β = −0.137, 95% CI −0.254 to −0.019; *p* = 0.022; Supplementary Table S3), trans-aconitate (β = −0.803, 95% CI −1.596 to −0.010; *p* = 0.047), and uracil (β = 0.903, 95% CI 0.049 to 1.757; *p* = 0.038). However, after FDR-correction for multiple comparisons, none of these associations remained statistically significant.

Interaction testing between each metabolite and sex, age, and BMI revealed evidence of effect modification by sex for pyroglutamate (*p* = 0.029) and taurine (*p* = 0.013). In subsequent sex-stratified analyses, shown in Table 3, significant associations between these metabolites and digit span performance were observed exclusively in men.

**Table 3:** Sex-stratified association of urinary metabolites with digit span (scaled score; WAIS-IV)

	β (95% CI)	*p*-value
Pyroglutamate		
Women	0.301 (−0.167; 0.769)	0.207
Men	−0.534 (−1.020; −0.048)	0.031*
Taurine		
Women	0.010 (−0.031; 0.052)	0.621
Men	−0.089 (−0.151; −0.027)	0.005*

*p < 0.05

Linear regression models were adjusted for age, sex, BMI, education level, physical activity, smoking status, alcohol consumption and cognition-related health conditions (psychiatric diseases, CVD and fatigue)

#### Verbal fluency

**Fig. 2:**
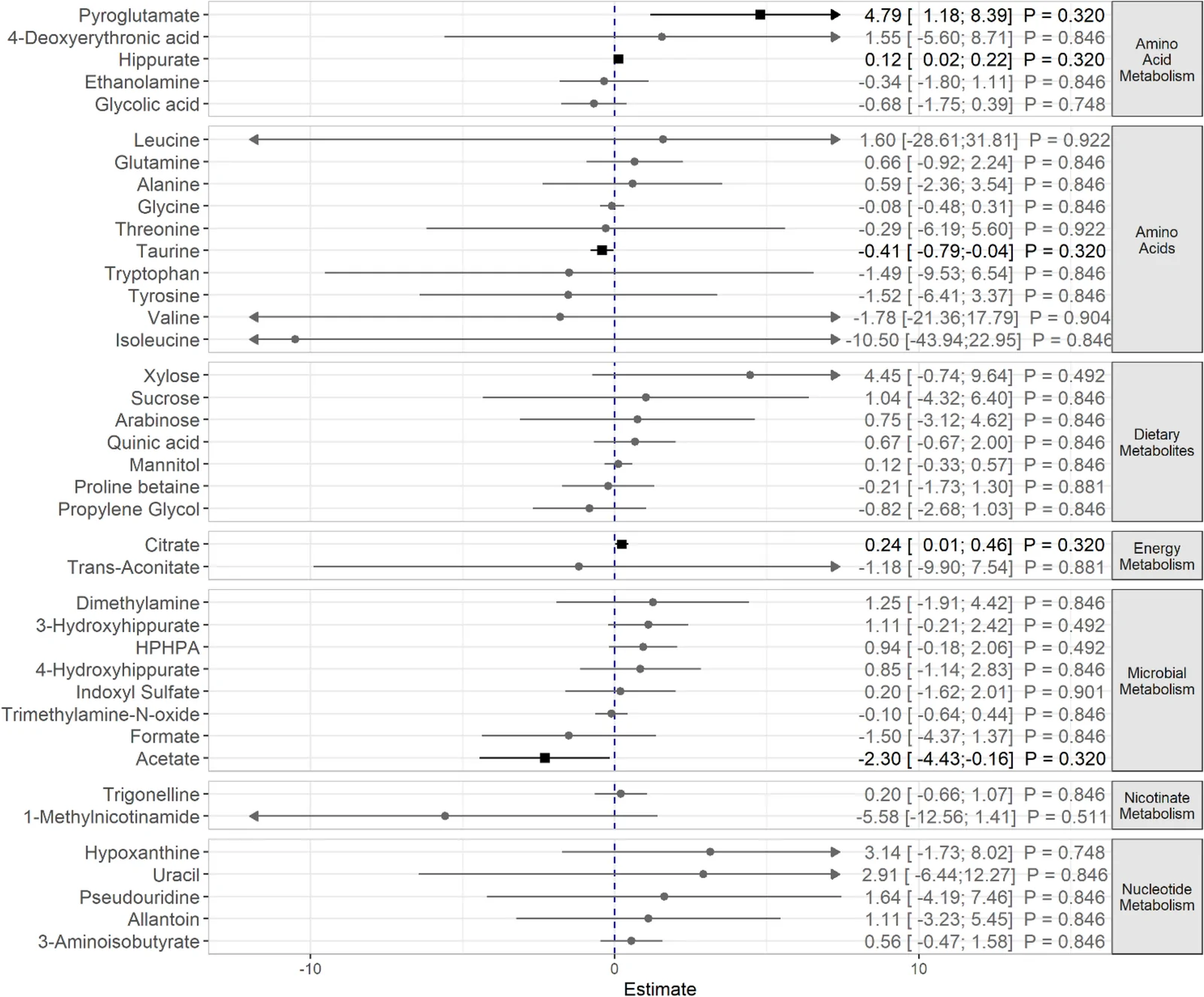
Associations between urinary metabolites and verbal fluency adjusted for age, sex, BMI, education level, physical activity level, smoking status, alcohol consumption, and cognition-related health conditions (psychiatric diseases, CVD and fatigue). Forest plots display regression coefficients and 95% confidence intervals. Corresponding *p*-values after FDR correction are presented on the right. Squares indicate associations that were statistically significant before FDR correction. Metabolites were grouped according to metabolic pathways. HPHPA: 3-(3-Hydroxyphenyl)-3-hydroxypropionic acid

**Fig. 3:**
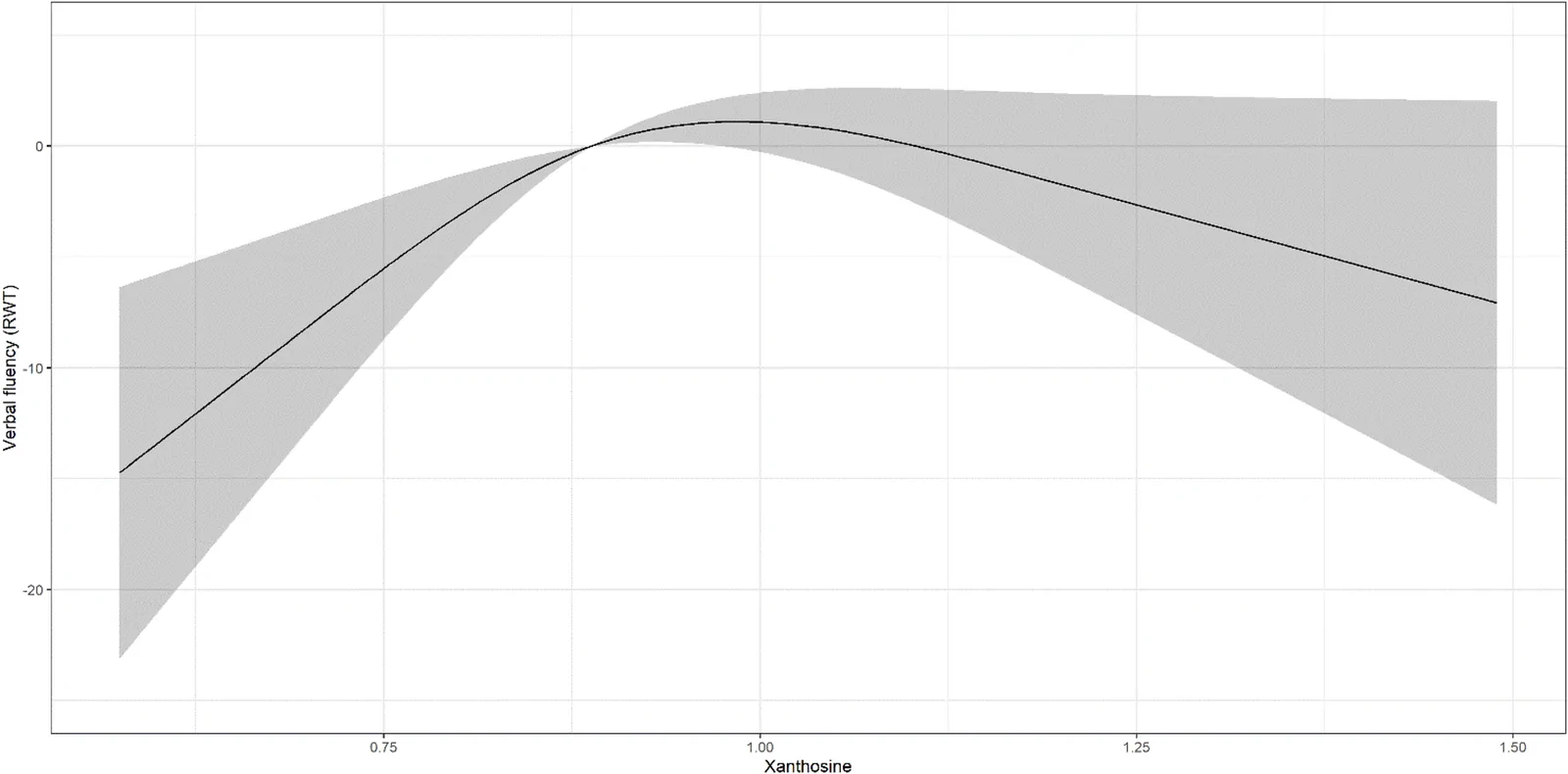
Restricted cubic spline illustrating the non-linear association between urinary xanthosine concentrations and verbal fluency (RWT), adjusted for age, sex, BMI, educational attainment, physical activity level, smoking status, alcohol consumption, and cognition-related health conditions (psychiatric disorders, CVD and fatigue). The solid line represents the estimated association, and the shaded area the 95% confidence band with the median xanthosine concentration as reference point

Our multivariable regression model revealed significant associations between six urinary metabolites and semantic verbal fluency, shown in Fig. 2, including acetate (β = −2.299, 95% CI − 4.433 to −0.164; *p* = 0.035), citrate (β = 0.237, 95% CI 0.010 to 0.464; *p* = 0.041; Supplementary Table S4), hippurate (β = 0.119, 95% CI 0.020 to 0.218; *p* = 0.018), pyroglutamate (β = 4.786, 95% CI 1.179 to 8.393; *p* = 0.009), taurine (β = −0.413, 95% CI −0.788 to −0.039; *p* = 0.030) and xanthosine (*p* = 0.002). These associations did not remain statistically significant after FDR correction. Supplementary Figure S1 shows restricted cubic spline models applied for metabolites that did not meet the linearity assumption. Among these, only xanthosine exhibited a significant overall inverse U-shaped association with the outcome in the spline-based model (Fig. 3, *p* = 0.002).

Interaction testing revealed an effect modification by sex for glutamine (*p* = 0.014) and propylene glycol (*p* = 0.007), but none by BMI or age. In sex-stratified analyses, shown in Table 4, glutamine was positively associated with verbal fluency in men, but not in women. In contrast, propylene glycol was inversely associated with verbal fluency in women, but not in men.

**Table 4:** Sex-stratified association of urinary metabolites with verbal fluency (RWT)

	β (95% CI)	*p*-value
Glutamine		
Women	−0.867 (−2.725; 0.992)	0.360
Men	2.951 (0.117; 5.784)	0.041*
Propylene glycol		
Women	−2.160 (−4.138; −0.182)	0.032*
Men	3.464 (−0.569; 7.497)	0.092

*p < 0.05

Linear regression models were adjusted for age, sex, BMI, education level, physical activity, smoking status, alcohol consumption and cognition-related health conditions (psychiatric diseases, CVD and fatigue)

## Discussion

In this analysis, urinary concentrations of 3-hydroxyhippurate, trans-aconitate, and uracil were associated with digit span performance, while acetate, citrate, hippurate, pyroglutamate, taurine, and xanthosine were associated with semantic verbal fluency. In addition, sex-specific associations were observed for pyroglutamate and taurine in relation to digit span, and for glutamine and propylene glycol in relation to verbal fluency. However, none of the observed associations remained statistically significant after correction for multiple testing. The following sections discuss these exploratory findings according to their primary metabolic pathways and potential relevance for cognitive function.

### Energy metabolism and TCA cycle

As a central intermediate of the tricarboxylic acid (TCA) cycle, citrate links glycolysis, fatty acid metabolism, and mitochondrial energy production (Akram 2014). In addition, it serves as an important source of acetyl-CoA, which is required for lipid synthesis and the production of acetylcholine, a neurotransmitter essential for cognitive function (Gibson and Shimada 1980). Existing studies have reported altered blood levels of citrate in cognition with heterogeneous results (Gutierrez-Tordera et al. 2024; Jaramillo-Jimenez et al. 2023; Zhang et al. 2022). Evidence on urinary citrate and cognition is scarce and limited to experimental metabolomic studies in dementia rat models revealing reduced urinary citrate concentrations, which is in line with our exploratory findings (Huang et al. 2022). Reduced citrate synthase activity and expression have been reported in AD with a potential contribution to impaired mitochondrial adenosine triphosphate (ATP) production, reduced acetyl-CoA and acetylcholine availability, and consequently impaired neuronal function (Chhimpa et al. 2023; Jia et al. 2023). In this context, the positive association between citrate and cognitive performance in our study may indicate a more efficient mitochondrial energy metabolism essential for cognitive functioning (Mukherjee et al. 2025).

In contrast, trans-aconitate, an isomer of the TCA cycle intermediate cis-aconitate, has been demonstrated to inhibit aconitase activity, thereby limiting the conversion of citrate to isocitrate (Saffran and Prado 1949). Its inverse association with working memory performance in our study may therefore point toward disruptions in mitochondrial energy metabolism, which is particularly relevant for the brain given its high energy demand (Bélanger et al. 2011). However, research addressing trans-aconitate in human health is limited and largely derived from studies linking it to inflammatory and metabolic conditions, such as ulcerative colitis or diabetic kidney disease, making its role in cognitive processes difficult to interpret (Braga et al. 2025; Keshteli et al. 2017).

Overall, these findings may indicate that TCA cycle-related metabolites reflect differences in mitochondrial energy metabolism that are potentially relevant for cognitive performance.

### Microbial and dietary metabolites

Hippurate is a well-established marker of gut microbial metabolism of dietary polyphenols, particularly from plant-based foods such as fruits and vegetables (Lees et al. 2013), and has been linked to microbial diversity, improved plasma glucose regulation and reduced metabolic syndrome risk (Pallister et al. 2017; Williams et al. 2010). In line with a potentially protective role, higher plasma hippurate levels have been associated with healthy aging based on NMR metabolomic analyses in a cohort of 269 individuals (Lawton et al. 2008), whereas lower urinary concentrations have been reported in individuals with MCI compared to healthy controls in a cohort of 59 geriatric patients (Yilmaz et al. 2020), which is consistent with the positive association with cognitive function in our study.

Interestingly, its metabolite 3-hydroxyhippurate showed an opposite association pattern in our study, being inversely associated with working memory performance. Although literature on 3-hydroxyhippurate and cognition remains sparse, this divergence may imply that closely related microbial metabolites might represent distinct aspects of host-microbiome metabolism with differing relevance for cognitive function. Supporting this, higher urinary 3-hydroxyhippurate levels were previously associated with fatigue within the same cohort (Kneipp et al. 2026), a condition frequently linked to cognitive impairments in attention and memory (Menzies et al. 2021).

Urinary acetate is primarily derived from gut microbial fermentation of carbohydrates (Moffett et al. 2020) and, unlike hippurate, has more frequently been implicated in metabolic conditions associated with high-calorie diets and metabolic syndrome (Perry et al. 2016), while few studies have investigated it in relation to cognitive function. Within the gut-brain axis, short-chain fatty acids such as acetate are thought to contribute to signaling between the gut microbiome and the central nervous system, suggesting a potential relevance for cognitive processes (Hosmer et al. 2024). Consistent with the inverse association between urinary acetate and verbal fluency observed in our study, Figueira et al. reported elevated salivary acetate concentrations in dementia patients compared with controls in an NMR analysis (Figueira et al. 2016). However, data across studies remain heterogeneous. Experimental studies in diabetic rats demonstrated that long-term acetate deficiency caused by depletion of acetate-producing bacteria impairs learning and memory (Zheng et al., 2021), while reduced acetate concentrations have also been identified in NMR studies in the CSF of 20 AD patients compared to 20 controls (Vignoli et al., 2020), and the serum of 21 AD patients and 28 controls (Di Costanzo et al., 2020).

Similar to acetate, propylene glycol has been linked to less favourable metabolic profiles of lower dietary quality and markers of metabolic dysregulation, including fasting glucose and insulin resistance and may imply diet- and lifestyle-related exposure patterns rather than endogenous metabolism (Trimigno et al. 2020). Propylene glycol is an exogenous compound commonly derived from food additives, pharmaceuticals, and cosmetic products (Jacob et al. 2018). Consistent with our findings, experimental evidence shows that propylene glycol may exert inhibitory effects on the central nervous system (Lin et al. 1998) and clinical studies have reported altered propylene glycol levels in the CSF of traumatic brain injury patients (Glenn et al. 2013) and in urine of sport-related concussion patients (Wanner et al. 2021). The female-specific association in our study may further point toward sex-dependent differences in exposure patterns, dietary behaviours, or xenobiotic metabolism.

These findings suggest that microbial metabolites associated with more favourable host metabolic profiles tend to relate to better cognitive performance, whereas metabolites linked to metabolic dysregulation or exogenous exposures show inverse associations with cognition.

### Oxidative stress and neurotransmission

Glutamine, a non-essential amino acid, plays a central role in the glutamatergic neurotransmission (Albrecht et al. 2010). Although evidence on urinary glutamine and cognition is lacking, previous studies using blood-based biomarkers have reported inconsistent associations, ranging from potentially protective effects in AD (Ramadan et al. 2024) to increased risk of incident dementia and worse cognitive performance (Cui et al. 2020; van der Lee et al. 2018). These divergent findings mirror the complex role of glutamatergic neurotransmission, in which both insufficient and excessive activity may contribute to dysfunction (Albrecht et al. 2010).

Taurine, a semi-essential amino acid, is involved in osmoregulation, energy metabolism, neurotransmission, and antioxidant defense (Schaffer and Kim 2018). Although numerous studies claim a neuroprotective role of taurine, findings remain inconsistent (Chen et al. 2019). Several studies have reported lower taurine levels in saliva (Figueira et al. 2016), urine (Yoshida et al. 2023), plasma and CSF (Basun et al. 1990) in patients with MCI or dementia compared to healthy controls, while others have found elevated urinary taurine levels in elderly individuals with dementia (Gao et al. 2017). This variability may indicate context- and domain-specific effects of taurine rather than uniformly beneficial associations with cognition. Experimental evidence further supports this interpretation, showing that taurine supplementation differentially affects cognitive performance across specific domains (Giles et al. 2012). Given taurine’s established role in oxidative defense, elevated urinary taurine may thus suggest a compensatory response to increased metabolic or oxidative stress rather than a direct detrimental effect on cognition.

Similarly, pyroglutamate, an intermediate of the gamma-glutamyl cycle involved in glutathione synthesis, may point toward alterations in glutathione turnover and redox homeostasis (Yu et al. 2002). Dickens et al. reported that higher CSF pyroglutamate concentrations in HIV patients were associated with impairments in global cognition, executive functioning, and verbal learning, whereas no associations were observed for verbal or working memory performance (Dickens et al. 2015), revealing domain-specific associations as in our study. In contrast, reduced pyroglutamate levels in serum and CSF have been linked to impaired cognitive states, including traumatic brain injury and AD (Trushina et al. 2013; Yi et al. 2016). Given the central role of glutathione as a major intracellular antioxidant, disturbances in glutathione metabolism have been implicated in neurodegenerative processes (Schulz et al. 2000). The heterogeneous associations observed for pyroglutamate in our study and in previous literature may consequently imply altered flux through the glutathione synthesis pathway and adaptive responses to varying oxidative demands.

The identified sex-specific associations may result from biological differences in amino acid metabolism between men and women, supported by prior literature on sex-dependent regulation of taurine homeostasis, potentially mediated by hormonal influences (Seghieri et al. 2025). Similarly, sex differences in glutathione metabolism may underlie the heterogeneous associations for pyroglutamate (Wang et al. 2020), while the male-specific association of glutamine with verbal fluency may correspond to previously described differences in glutamatergic system regulation at multiple levels, including receptor expression and metabolic control (Wickens et al. 2018).

### Nucleotide metabolism and oxidative stress

**Uracil** is a pyrimidine base and precursor of uracil-containing nucleotides, which play essential roles in RNA synthesis and cellular metabolic processes, including glycosylation and glycogen metabolism. Beyond its structural functions, uracil nucleotides are also involved in CNS signaling pathways with both neuroprotective and neuroinflammatory properties (Lecca and Ceruti 2008). Although available research discussing uracil in the context of cognitive function is scarce, reduced circulating levels of its nucleoside form uridine have been associated with clinical progression of Alzheimer’s disease, suggesting a potential involvement in neurodegenerative processes (de Leeuw et al. 2020).

In contrast, xanthosine is a purine-derived nucleoside involved in purine degradation pathways closely linked to oxidative stress regulation. Through downstream metabolism via xanthine oxidoreductase (XOR), purine catabolism contributes to the generation of reactive oxygen species, promoting inflammatory processes and endothelial dysfunction, mechanisms implicated in cognitive decline (Bortolotti et al. 2021; Franzoni et al. 2021). Experimental evidence further supports a role of XOR activation in stress-induced oxidative damage, cerebrovascular dysfunction, and impaired working memory in animal models (Burrage et al. 2023; Prabhu et al. 2023). The non-linear, inverse U-shaped association observed in our study may indicate that both low and high levels of purine turnover might represent metabolically unfavourable states in relation to cognitive performance.

### Strengths and limitations

This study has several strengths. The use of urinary metabolomics provides a non-invasive approach to assess metabolic profiles, making it feasible for large-scale population studies. The sample size was substantial, allowing for robust statistical analyses and we were able to adjust for a comprehensive set of potential confounders based on extensive participant data on demographics, lifestyle factors, and health conditions. Finally, cognitive performance was assessed using two standardized and well-validated tests of executive function, digit span and semantic verbal fluency, ensuring reliable measurement of key cognitive domains.

However, several limitations should be acknowledged. The study is exploratory and hypothesis-generating, and its cross-sectional design precludes causal inference and limits conclusions to associations. None of our results remained significant after FDR correction, however, this procedure is not necessarily used in explorative studies. While urine is a convenient and non-invasive biospecimen, the urinary metabolome is narrower than that found in blood or CSF, being largely free from proteins and lipids due to glomerular filtration, and is subject to variability from diet, microbiome composition, and kidney function (Bouatra et al. 2013). Individual differences in lifestyle and environmental exposures may further influence metabolite profiles. The sample primarily included adults aged 18–75 years from a single geographic area, limiting generalizability to older populations or other ethnic groups.

## Conclusion

Taken together, our exploratory findings suggest that urinary metabolites related to energy, microbial, amino acid, and nucleotide metabolism may be associated with domain- and sex-specific differences in cognitive performance. Our results highlight the potential of urinary metabolomics to capture integrated biological processes that may be relevant to early cognitive variation in the general population. However, future research should be done in studies with longitudinal study design, with larger sample size, and diverse participants to confirm and further clarify the observed metabolite-cognition associations.

## Supplementary Information

Below is the link to the electronic supplementary material.

Supplementary Material 1 (PDF 477 KB)

## Data Availability

The data underlying this article cannot be shared publicly because the data are subject to national data protection laws and restrictions that were imposed by the ethics committee of the Ludwig-Maximilians University, Munich, to ensure data privacy of the study participants because they did not explicitly consent to the data being made publicly available. The data will be shared at reasonable requests to the corresponding.

## Abbreviations

ATP: Adenosine triphosphate
AUDIT: Alcohol Use Disorders Identification Test
BMI: Body Mass Index
CSF: Cerebrospinal fluid
CVD: Cardiovascular disease
EHIS-PAQ: European Health Interview Survey-Physical Activity Questionnaire
FAS: Fatigue Assessment Scale
FDR: False discovery rate
^1^H NMR: Proton nuclear magnetic resonance spectroscopy
ISCED: International Standard Classification of Education
MCI: Mild cognitive impairment
MEIA: Metabolism, Nutrition, and Immune System in Augsburg Study
RWT: Regensburger Wortflüssigkeitstest
TCA: Tricarboxylic acid
WAIS-IV: Wechsler Adult Intelligence Scale IV
WMI: Working Memory Index
XOR: Xanthine oxidoreductase
