# Supplementary material for: Urinary metabolomics data and cognitive performance in an adult population
Source: Metab Brain Dis. 2026 Sep 12;41(1):218. doi: 10.1007/s11011-026-01977-8 (PMC13570828; doi:10.1007/s11011-026-01977-8)
Supplement: Supplementary file 1 [file 11011_2026_1977_MOESM1_ESM.pdf]

Supplement

Metabolomics

Urinary Metabolomics Data and Cognitive Function in an Adult Population

Annika Kneipp[1], Inge Kirchberger[1], Dennis Freuer[1], Christine Meisinger[1], Jakob Linseisen[1]

[1] *Epidemiology, Faculty of Medicine, University of Augsburg, Augsburg, Germany*

*Corresponding author:
Prof. Dr. Jakob Linseisen
Institute of Epidemiology, University Hospital Augsburg
Stenglinstr. 2
86156 Augsburg,
Germany
Tel.: +49-821-5986471
Email: jakob.linseisen@med.uni-augsburg.de

Table S1 Baseline characteristics of participants of the population-based MEIA study, stratified by digit span (WAIS-IV, scaled score)

	Total (n = 560)	Below Average (n = 72)	Average (n = 454)	Above Average (n = 34)	*p*-values	N*
Median (Q25, Q75)						
**Age [years]**	49.0 (34.0; 60.0)	46.5 (33.0; 57.0)	48.0 (34.0; 60.0)	57.0 (48.8; 64.3)	< 0.001	560
**BMI [kg/m^2]**	25.7 (22.8; 29.0)	25.5 (23.0; 29.3)	25.7 (22.6; 28.9)	26.9 (22.9; 30.7)	0.749	559
**Verbal fluency (RWT)**	77 (48; 94)	43 (12; 69)	79 (52; 95)	96 (80; 99)	< 0.001	553
n (%)						
**Sex**					0.922	560
Female	314 (56.1 %)	40 (55.6 %)	256 (56.4 %)	18 (52.9 %)		
Male	246 (43.9 %)	32 (44.4 %)	198 (43.6 %)	16 (47.1 %)		
**Age goups**					0.007	560
18-39 years	193 (34.5 %)	28 (38.9 %)	162 (35.7 %)	3 (8.8 %)		
40-60 years	232 (41.4 %)	31 (43.1 %)	185 (40.7 %)	16 (47.1 %)		
61-75 years	135 (24.1 %)	13 (18.1 %)	107 (23.6 %)	15 (44.1 %)		
**Education level**					0.243	560
ISCED 1-3	112 (20.0 %)	15 (20.8 %)	94 (20.7 %)	3 (8.8 %)		
ISCED 4-6	448 (80.0 %)	57 (79.2 %)	360 (79.3 %)	31 (91.2 %)		
**Physical activity**					0.110	556
Sedentary	108 (19.4 %)	12 (16.7 %)	90 (20.0 %)	6 (17.6 %)		
Low Active	169 (30.4 %)	19 (26.4 %)	139 (30.9 %)	11 (32.4 %)		
Active	157 (28.2 %)	15 (20.8 %)	133 (29.6 %)	9 (26.5 %)		
Very Active	122 (21.9 %)	26 (36.1 %)	88 (19.6 %)	8 (23.5 %)		
**Smoking status**					0.992	560
Current	94 (16.8 %)	13 (18.1 %)	75 (16.5 %)	6 (17.6 %)		
Never	275 (49.1 %)	36 (50.0 %)	223 (49.1 %)	16 (47.1 %)		
Previous	191 (34.1 %)	23 (31.9 %)	156 (34.4 %)	12 (35.3 %)		
**Risky alcohol consumption**					0.144	557
Low	270 (48.5 %)	42 (59.2 %)	212 (46.9 %)	16 (47.1 %)		
Moderate	211 (37.9 %)	19 (26.8 %)	182 (40.3 %)	10 (29.4 %)		
High	54 (9.7 %)	6 (8.5 %)	43 (9.5 %)	5 (14.7 %)		
Severe	22 (3.9 %)	4 (5.6 %)	15 (3.3 %)	3 (8.8 %)		
**Psychiatric disorder**					0.619	557
Yes	55 (9.9 %)	7 (9.9 %)	43 (9.5 %)	5 (14.7 %)		
No	502 (90.1 %)	64 (90.1 %)	409 (90.5 %)	29 (85.3 %)		
**CVD**					0.118	558
Yes	19 (3.4 %)	5 (7.0 %)	12 (2.6 %)	2 (5.9 %)		
No	539 (96.6 %)	66 (93.0 %)	441 (97.4 %)	32 (94.1 %)		
**Fatigue**					0.209	553
Yes (FAS ≥ 22)	129 (23.3 %)	19 (27.1 %)	106 (23.6 %)	4 (11.8 %)		
No (FAS < 22)	424 (76.7 %)	51 (72.9 %)	343 (76.4 %)	30 (88.2 %)		

Verbal fluency (RWT)					< 0.001	553
Below average	57 (10.3 %)	18 (26.1 %)	39 (8.6 %)	0 (0.0 %)		
Average	263 (47.6 %)	39 (56.5 %)	214 (47.5 %)	10 (30.3 %)		
Above average	233 (42.1 %)	12 (17.4 %)	198 (43.9 %)	23 (69.7 %)		

*Number of cases with valid information

P-values were derived from Kruskal-Wallis tests for continuous variables and Chi-Square tests for categorical variables.

Psychiatric disorders include self-reported depression, anxiety, and psychosis.
CVD include major cardiovascular diseases, such as stroke and myocardial infarction.

Table S2 Creatinine-corrected urinary metabolite concentrations [mmol/mmol creatinine x 100] in the total study population and stratified by digit span performance groups (scaled score, WAIS-IV)

	**Total (*n* = 560)**	**Below Average (n = 72)**	**Average (n = 454)**	**Above Average (n = 34)**	***p*-values[a]**
Acetate	0.466 (0.263; 0.794)	0.452 (0.245; 0.789)	0.467 (0.270; 0.799)	0.472 (0.164; 0.665)	0.636
Alanine	1.752 (1.331; 2.254)	1.803 (1.366; 2.519)	1.721 (1.325; 2.210)	1.844 (1.341; 2.295)	0.559
Allantoin	0.543 (0.269; 0.968)	0.497 (0.274; 0.882)	0.564 (0.286; 0.993)	0.370 (0.185; 0.910)	0.277
2-Hydroxyisobutyrate	0.495 (0.402; 0.592)	0.499 (0.374; 0.583)	0.495 (0.403; 0.592)	0.480 (0.438; 0.618)	0.875
Arabinose	0.446 (0.298; 0.647)	0.440 (0.317; 0.669)	0.447 (0.296; 0.645)	0.449 (0.258; 0.572)	0.898
3-Aminoisobutyrate	0.489 (0.159; 1.144)	0.409 (0.173; 1.325)	0.498 (0.159; 1.036)	0.614 (0.188; 1.366)	0.732
3-Hydroxyisobutyrate	0.683 (0.519; 0.874)	0.670 (0.529; 0.837)	0.677 (0.515; 0.878)	0.758 (0.586; 0.836)	0.499
Cis-Aconitate	1.726 (1.386; 2.227)	1.777 (1.282; 2.130)	1.713 (1.408; 2.240)	1.858 (1.196; 2.284)	0.604
Citrate	19.335 (12.075; 29.094)	18.350 (11.990; 29.137)	19.441 (11.833; 28.874)	20.465 (12.771; 33.446)	0.703
Dimethylamine	2.970 (2.709; 3.328)	2.922 (2.681; 3.337)	2.960 (2.711; 3.305)	3.087 (2.716; 3.428)	0.444
4-Deoxyerythronic acid	0.680 (0.527; 0.910)	0.697 (0.551; 0.922)	0.674 (0.517; 0.897)	0.768 (0.663; 1.139)	0.083
4-Deoxythreonate	2.094 (1.546; 2.716)	2.009 (1.482; 2.425)	2.113 (1.547; 2.743)	2.013 (1.687; 2.632)	0.473
Ethanolamine	4.030 (3.024; 5.066)	4.075 (3.163; 4.991)	4.045 (3.008; 5.089)	3.642 (2.700; 4.590)	0.475
Formate	1.515 (0.998; 2.007)	1.633 (1.086; 2.221)	1.476 (0.997; 1.975)	1.560 (0.753; 2.317)	0.568
Glutamine	2.037 (1.139; 3.163)	2.066 (1.396; 3.010)	1.996 (1.114; 3.198)	2.058 (0.879; 3.376)	0.964
Glycine	8.042 (5.534; 11.463)	8.272 (6.233; 11.345)	8.029 (5.450; 11.763)	7.710 (5.952; 9.186)	0.509
Glycolic acid	3.894 (2.751; 5.243)	3.610 (2.411; 5.477)	3.942 (2.769; 5.244)	3.563 (2.075; 4.516)	0.386
Hippurate	23.593 (14.759; 38.935)	24.955 (14.918; 42.120)	22.685 (14.439; 38.861)	27.855 (19.667; 38.080)	0.401
3-(3-Hydroxyphenyl)-3-hydroxypropionic acid	1.461 (0.483; 3.063)	1.561 (0.538; 2.908)	1.464 (0.483; 3.200)	1.327 (0.034; 2.247)	0.319
Hypoxanthine	0.823 (0.592; 1.121)	0.779 (0.594; 1.071)	0.823 (0.590; 1.121)	0.911 (0.620; 1.177)	0.841

Isoleucine	0.086 (0.055; 0.139)	0.084 (0.050; 0.135)	0.088 (0.056; 0.141)	0.070 (0.052; 0.120)	0.524
Indoxyl Sulfate	2.528 (1.785; 3.477)	2.577 (1.769; 3.542)	2.480 (1.722; 3.476)	2.944 (2.382; 3.445)	0.070
Lactate	0.900 (0.586; 1.531)	0.903 (0.608; 1.593)	0.899 (0.572; 1.531)	0.997 (0.641; 1.407)	0.941
Leucine	0.173 (0.134; 0.225)	0.201 (0.140; 0.267)	0.169 (0.132; 0.218)	0.185 (0.154; 0.232)	0.038*
Mannitol	1.057 (0.416; 3.179)	0.899 (0.379; 2.551)	1.086 (0.412; 3.253)	1.006 (0.543; 4.860)	0.412
3-Hydroxyhippurate	1.452 (0.731; 2.925)	1.513 (0.925; 3.108)	1.442 (0.679; 2.955)	1.561 (0.784; 2.607)	0.289
1-Methylnicotinamide	0.619 (0.459; 0.841)	0.577 (0.419; 0.828)	0.631 (0.463; 0.843)	0.604 (0.417; 0.818)	0.484
Pyroglutamate	2.249 (1.919; 2.665)	2.337 (2.002; 2.696)	2.216 (1.881; 2.641)	2.346 (1.956; 2.857)	0.194
4-Hydroxyhippurate	1.056 (0.733; 1.557)	1.040 (0.744; 1.717)	1.061 (0.735; 1.556)	0.879 (0.651; 1.503)	0.489
Propylene glycol	0.366 (0.185; 0.614)	0.359 (0.202; 0.655)	0.368 (0.187; 0.610)	0.311 (0.164; 0.598)	0.862
Proline betaine	0.636 (0.305; 1.491)	0.610 (0.374; 1.673)	0.641 (0.291; 1.467)	0.566 (0.297; 1.418)	0.411
Pseudouridine	3.029 (2.741; 3.323)	2.996 (2.718; 3.412)	3.024 (2.740; 3.310)	3.120 (2.876; 3.339)	0.486
Quinic acid	2.215 (1.060; 3.765)	1.578 (0.716; 2.942)	2.338 (1.113; 3.872)	2.107 (1.387; 3.322)	0.113
Sucrose	0.051 (0.000; 0.228)	0.052 (0.000; 0.190)	0.050 (0.000; 0.237)	0.057 (0.000; 0.229)	0.897
Trans-Aconitate	0.433 (0.323; 0.547)	0.430 (0.324; 0.533)	0.435 (0.319; 0.556)	0.426 (0.335; 0.507)	0.770
Taurine	2.843 (0.570; 6.435)	4.084 (0.656; 7.937)	2.771 (0.525; 6.164)	2.305 (0.809; 5.873)	0.326
Threonine	0.585 (0.388; 0.834)	0.671 (0.402; 0.916)	0.584 (0.391; 0.822)	0.550 (0.342; 0.988)	0.469
Trimethylamine-N-oxide	3.543 (2.496; 5.157)	4.055 (2.399; 5.522)	3.459 (2.482; 5.070)	3.646 (3.005; 5.366)	0.163
Trigonelline	2.896 (1.463; 4.679)	2.471 (1.222; 5.151)	2.959 (1.526; 4.658)	2.446 (1.503; 4.053)	0.564
Tryptophan	0.561 (0.408; 0.744)	0.549 (0.407; 0.734)	0.563 (0.411; 0.746)	0.541 (0.383; 0.681)	0.768
Tyrosine	0.937 (0.653; 1.287)	0.954 (0.723; 1.234)	0.937 (0.650; 1.273)	0.874 (0.594; 1.330)	0.939
Uracil	0.518 (0.389; 0.680)	0.480 (0.383; 0.680)	0.520 (0.388; 0.675)	0.550 (0.435; 0.755)	0.443
Urea	3218.766 (2408.012; 4113.006)	3086.689 (2311.075; 3885.735)	3241.584 (2436.724; 4118.812)	2967.107 (2053.294; 4391.228)	0.782
Valine	0.217 (0.166; 0.276)	0.219 (0.167; 0.278)	0.217 (0.164; 0.275)	0.211 (0.191; 0.282)	0.884
Xanthosine	0.888 (0.784; 1.017)	0.859 (0.770; 1.047)	0.893 (0.784; 1.015)	0.888 (0.811; 0.999)	0.809
Xylose	0.606 (0.354; 0.807)	0.668 (0.451; 0.830)	0.590 (0.334; 0.794)	0.638 (0.423; 0.915)	0.130

* $p < 0.05$

[a] Kruskal-Wallis Test

Table S3 Associations of urinary metabolites with digit span performance (scaled score; WAIS-IV)

	**β (95% CI)**	***p*-value**
Acetate	-0.146 (-0.341; 0.048)	0.139
Alanine	-0.089 (-0.359; 0.182)	0.520
Allantoin	-0.103 (-0.498; 0.292)	0.609
2-Hydroxyisobutyrate	0.153 (-1.412; 1.719)	0.847

Arabinose	-0.193 (-0.548; 0.161)	0.285
3-Aminoisobutyrate	0.021 (-0.072; 0.114)	0.659
3-Hydroxyisobutyrate	0.085 (-0.540; 0.710)	0.790
Cis-Aconitate	-0.169 (-0.428; 0.090)	0.200
Citrate	0.020 (-0.001; 0.041)	0.065
Dimethylamine	0.124 (-0.166; 0.414)	0.402
4-Deoxyerythronic acid	0.369 (-0.287; 1.024)	0.270
4-Deoxythreonate	0.117 (-0.112; 0.347)	0.316
Ethanolamine	0.011 (-0.122; 0.145)	0.868
Formate	-0.136 (-0.399; 0.127)	0.310
Glutamine	-0.009 (-0.154; 0.136)	0.904
Glycine	-0.019 (-0.055; 0.017)	0.302
Glycolic acid	-0.023 (-0.121; 0.074)	0.639
Hippurate	-0.003 (-0.012; 0.006)	0.538
3-(3-Hydroxyphenyl)-3-hydroxypropionic acid	-0.093 (-0.195; 0.010)	0.077
Hypoxanthine	0.263 (-0.184; 0.710)	0.248
Isoleucine	0.217 (-2.848; 3.283)	0.889
Indoxyl Sulfate	-0.048 (-0.214; 0.119)	0.573
Lactate	0.021 (-0.118; 0.160)	0.768
Leucine	-1.045 (-3.810; 1.721)	0.458
Mannitol	-0.011 (-0.052; 0.031)	0.617
3-Hydroxyhippurate	-0.147 (-0.267; -0.027)	0.016*
1-Methylnicotinamide	-0.120 (-0.759; 0.520)	0.714
Pyroglutamate	-0.141 (-0.473; 0.191)	0.403
4-Hydroxyhippurate	-0.100 (-0.282; 0.082)	0.281
Propylene glycol	0.006 (-0.164; 0.177)	0.941
Proline betaine	-0.053 (-0.192; 0.086)	0.454
Pseudouridine	0.056 (-0.478; 0.590)	0.837
Quinic acid	-0.023 (-0.145; 0.100)	0.714
Sucrose	0.343 (-0.149; 0.834)	0.171
Trans-Aconitate	-0.803 (-1.596; -0.010)	0.047*
Taurine	-0.025 (-0.060; 0.009)	0.153
Threonine	-0.352 (-0.892; 0.187)	0.200
Trimethylamine-N-oxide	0.011 (-0.038; 0.060)	0.658
Trigonelline	-0.040 (-0.119; 0.039)	0.323
Tryptophan	-0.035 (-0.772; 0.701)	0.926
Tyrosine	0.133 (-0.315; 0.582)	0.559
Uracil	0.903 (0.049; 1.757)	0.038*
Urea	0.000 (-0.000; 0.000)	0.462
Valine	0.090 (-1.709; 1.889)	0.922
Xanthosine	-0.019 (-1.093; 1.056)	0.973

Xylose	0.005 (-0.472; 0.481)	0.985

Linear regression model adjusted for age, sex, BMI, education level, physical activity level, smoking status, alcohol consumption and cognition-related health conditions (psychiatric diseases, CVD and fatigue), before FDR correction; p-values are displayed with * indicating $p < 0.05$.

Table S4 Associations of urinary metabolites with verbal fluency performance (RWT)

	**β (95% CI)**	***p*-value**
Acetate	-2.299 (-4.433; -0.164)	0.035*
Alanine	0.593 (-2.359; 3.545)	0.693
Allantoin	1.109 (-3.233; 5.452)	0.616
Arabinose	0.749 (-3.116; 4.615)	0.703
3-Aminoisobutyrate	0.557 (-0.468; 1.582)	0.286
Citrate	0.237 (0.010; 0.464)	0.041*
Dimethylamine	1.253 (-1.909; 4.416)	0.437
4-Deoxyerythronic acid	1.554 (-5.599; 8.707)	0.670
Ethanolamine	-0.344 (-1.800; 1.112)	0.643
Formate	-1.501 (-4.370; 1.369)	0.305
Glutamine	0.662 (-0.921; 2.245)	0.412
Glycine	-0.084 (-0.479; 0.311)	0.677
Glycolic acid	-0.679 (-1.745; 0.387)	0.211
Hippurate	0.119 (0.020; 0.218)	0.018*
3-(3-Hydroxyphenyl)-3-hydroxypropionic acid	0.936 (-0.183; 2.055)	0.101
Hypoxanthine	3.145 (-1.729; 8.018)	0.206
Isoleucine	-10.500 (-43.945; 22.946)	0.538
Indoxyl Sulfate	0.196 (-1.620; 2.013)	0.832
Leucine	1.600 (-28.612; 31.812)	0.917
Mannitol	0.121 (-0.331; 0.572)	0.599
3-Hydroxyhippurate	1.107 (-0.207; 2.421)	0.099
1-Methylnicotinamide	-5.575 (-12.560; 1.411)	0.118
Pyroglutamate	4.786 (1.179; 8.393)	0.009*
4-Hydroxyhippurate	0.847 (-1.138; 2.831)	0.402
Propylene glycol	-0.825 (-2.681; 1.031)	0.383
Proline betaine	-0.214 (-1.728; 1.300)	0.782
Pseudouridine	1.638 (-4.190; 7.465)	0.581
Quinic acid	0.666 (-0.673; 2.005)	0.329
Sucrose	1.036 (-4.325; 6.398)	0.704
Trans-Aconitate	-1.177 (-9.900; 7.545)	0.791
Taurine	-0.413 (-0.788; -0.039)	0.030*
Threonine	-0.293 (-6.186; 5.600)	0.922
Trimethylamine-N-oxide	-0.103 (-0.641; 0.435)	0.706
Trigonelline	0.204 (-0.658; 1.066)	0.642

Tryptophan	-1.491 (-9.525; 6.542)	0.716
Tyrosine	-1.518 (-6.407; 3.370)	0.542
Uracil	2.911 (-6.445; 12.267)	0.541
Valine	-1.784 (-21.357; 17.790)	0.858
Xylose	4.454 (-0.735; 9.642)	0.092

Linear regression model adjusted for age, sex, BMI, education level, physical activity level, smoking status, alcohol consumption and cognition-related health conditions (psychiatric diseases, CVD and fatigue), before FDR correction; p-values are displayed with * indicating $p < 0.05$.

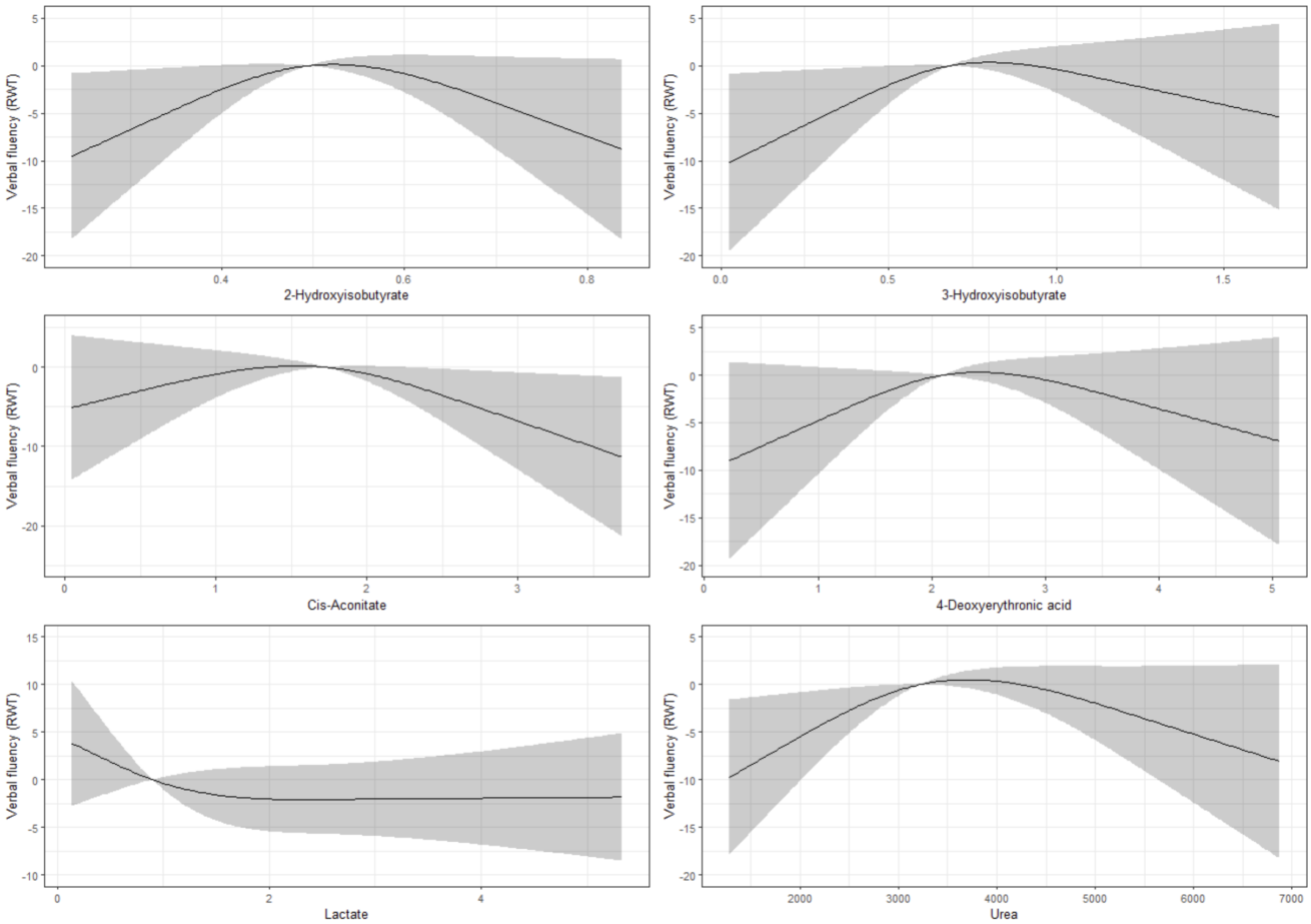

Fig. S1 Restricted cubic spline illustrating the non-linear associations between urinary concentrations and verbal fluency (RWT), adjusted for age, sex, BMI, educational attainment, physical activity, smoking status, alcohol consumption, and cognition-related health conditions (psychiatric disorders, CVD and fatigue). The solid line represents the estimated association, and the shaded area the 95% confidence band with the median xanthosine concentration as reference point
